# Proficiency testing of PIK3CA mutations in HR+/HER2-breast cancer on liquid biopsy and tissue

**DOI:** 10.1007/s00428-022-03445-x

**Published:** 2022-11-11

**Authors:** Claudia Vollbrecht, Inga Hoffmann, Annika Lehmann, Sabine Merkelbach-Bruse, Jana Fassunke, Svenja Wagener-Ryczek, Markus Ball, Lora Dimitrova, Arndt Hartmann, Robert Stöhr, Ramona Erber, Wilko Weichert, Nicole Pfarr, Lisa Bohlmann, Andreas Jung, Wolfgang Dietmaier, Manfred Dietel, David Horst, Michael Hummel

**Affiliations:** 1 Charité-Universitätsmedizin Berlin, Corporate Member of Freie Universität Berlin, Humboldt-Universität zu Berlin and Berlin Institute of Health, Institute of Pathology, Berlin, Germany; 2 Faculty of Medicine and University Hospital Cologne, Institute of Pathology, University of Cologne, Cologne, Germany; 3 Quality in Pathology (QuIP GmbH), Berlin, Germany; 4 Institute of Pathology, University Hospital Erlangen, Friedrich-Alexander-Universität Erlangen-Nürnberg (FAU), Comprehensive Cancer Center Erlangen-EMN, 91054 Erlangen, Germany; 5 Institute of Pathology, Technical University Munich, Munich, Germany; 6 Pathologisches Institut of the Ludwig-Maximilian-Universität München, Munich, Germany; 7 German Cancer Consortium (DKTK), Partner Site Munich, Munich, Germany; 8 Institute of Pathology, University of Regensburg, Regensburg, Germany

**Keywords:** PIK3CA, Breast cancer, Liquid biopsy, Proficiency testing, Alpelisib

## Abstract

Precision oncology based on specific molecular alterations requires precise and reliable detection of therapeutic targets in order to initiate the optimal treatment. In many European countries—including Germany—assays employed for this purpose are highly diverse and not prescribed by authorities, making inter-laboratory comparison difficult. To ensure reproducible molecular diagnostic results across many laboratories and different assays, ring trials are essential and a well-established tool. Here, we describe the design and results of the ring trial for the detection of therapeutically relevant *PIK3CA* hotspot mutations in HR+/HER2-breast cancer tissue and liquid biopsy (LB). For *PIK3CA* mutation detection in tissue samples, 43 of the 54 participants (80%) provided results compliant with the reference values. Participants using NGS-based assays showed higher success rate (82%) than those employing Sanger sequencing (57%). LB testing was performed with two reference materials differing in the length of the mutated DNA fragments. Most participants used NGS-based or commercial real-time PCR assays (70%). The 167 bp fragments led to a successful *PIK3CA* mutation detection by only 31% of participants whereas longer fragments of 490 bp were detectable even by non-optimal assays (83%). In conclusion, the first ring trial for *PIK3CA* mutation detection in Germany showed that *PIK3CA* mutation analysis is broadly established for tissue samples and that NGS-based tests seem to be more suitable than Sanger sequencing. *PIK3CA* mutation detection in LB should be carried out with assays specifically designed for this purpose in order to avoid false-negative results.

**Supplementary Information:**

The online version contains supplementary material available at 10.1007/s00428-022-03445-x.

## Introduction

Breast cancer is the most common malignancy and the second most cancer-related cause of death in women in the USA [1]. While testing of targeted therapy-relevant biomarkers is already standard for other cancer types like melanoma, lung, and colon cancer, the use of companion diagnostics for breast cancer was so far limited to the analysis of progesterone/estrogen receptors, human epidermal growth factor receptor 2 (*HER2*) and programmed cell death 1 ligand 1 (PD-L1) as well as to the analysis of germline alterations of *BRCA1* and *BRCA2* genes for inherited disease [2]. The vast majority of breast cancer patients are hormone receptor-positive (HR+) and HER2-negative (HER2-) [3, 4]. Currently, the standard therapy for HR+/HER2-breast cancer patients is treated with aromatase inhibitors (e.g., anastrazole, letrozole, or exemestane) alone or in combination with cyclin-dependent kinase 4/6 inhibitors (e.g., ribociclib, palbociclib, or abemaciclib) [5]. However, these patients are often faced with the challenge of therapy resistance. Therefore, data from the GENIE-Project (Genomics Evidence Neoplasia Information Exchange) of the American Association for Cancer Research (AACR) showing that *PIK3CA* is the most frequent mutated gene in HR+/HER2-breast cancer patients, are very promising [6, 7]. The *PIK3CA* gene encodes the ⍺-isoform p110 of the catalytic subunit of the phosphatidylinositol-3-kinase (PI3K), which plays a major role in PI3K/PTEN/AKT signaling. Its activation via extracellular stimuli like growth factors, cytokines, or hormones catalyzes the transformation from PIP2 (phosphatidylinositol-4,5-bisphosphat) to PIP3 (phosphatidylinositol-3,4,5-trisphosphate) and leads to the intracellular activation of downstream processes for cell growth, proliferation, mortality, and angiogenesis [8].

Activating mutations in the *PIK3CA* gene lead to a hyperactivation of the catalytic subunit p110α and may lead to a dysregulation of downstream PI3K signaling [9, 10]. Mutation hotspots are found in the codons 542, 545, and 1047, making *PIK3CA* an auspicious biomarker for targeted therapy approaches using selective p110α inhibitors [11, 12].

In the SOLAR-1 phase III trial, *PIK3CA* mutated HR+/HER2-breast cancer patients clearly benefit from a therapy with the α-specific PIK3CA inhibitor alpelisib [13]. After receiving an endocrine therapy, *PIK3CA* mutated HR+/HER2-breast cancer patients treated with alpelisib plus fulvestrant showed a median progression-free survival (PFS) of 11 months vs. 5.7 months for patients treated with placebo plus fulvestrant (*HR* = 0.65, 95% CI: 0.50, 0.85, one-sided *p*-value = 0.00065) [13]. Hence, alpelisib was approved by the U.S. Food and Drug Administration (FDA) in 2019 making it the first PI3K inhibitor for breast cancer patients. The molecular stratification of patients based on activating *PIK3CA* mutations found in tissue and for the first time also in liquid biopsies (LB), was performed by quantitative PCR (qPCR) assay therascreen PIK3CA RGQ (Qiagen, Hilden, Germany) [13]. The European Medicines Agency (EMA) approval followed in 2020 for the same patient group using a validated test for the analysis of *PIK3CA* mutations. Based on these requirements the first independent proficiency testing for *PIK3CA* mutations in tissue and LB samples was initiated by Quality in Pathology-QuIP GmbH in 2020.

While testing formalin-fixed paraffin-embedded (FFPE) tissue is well established, mutation detection in cell-free DNA (cfDNA) from clinical plasma samples is more challenging due to the low amount of circulating tumor DNA (ctDNA). In addition, a relatively large volume of peripheral blood is required per patient, making it less suitable for proficiency testing. To receive a realistic evaluation of the eligibility of methods used in the diagnostic setting, appropriate reference material is required. Therefore, two types of defined customized commercial reference samples (SensID, Rostock, Germany) were employed for the first time in a QuIP proficiency test to simulate cfDNA and ctDNA conditions present in clinical plasma samples.

The aim of the proficiency testing scheme “*PIK3CA* mutations in HR+/HER2-breast cancer” was the quality assessment of validated, correctly performed, and reproducible *PIK3CA* mutation testing by the participating institutes without methodological restrictions including locally validated laboratory-developed tests (LDTs), as well as research use only (RUO) and CE-IVD (CE in vitro diagnostics) assays. The ring trial was open to all laboratories to obtain qualification for *PIK3CA* testing and ring trial results can be used for accreditation.

Thus, an overview of the healthcare landscape of *PIK3CA* mutation testing in breast cancer on tissue and LB material using molecular pathology methods in the German-speaking regions was obtained.

## Material and methods

### Case selection and study design

#### Tissue

**Fig 1:**
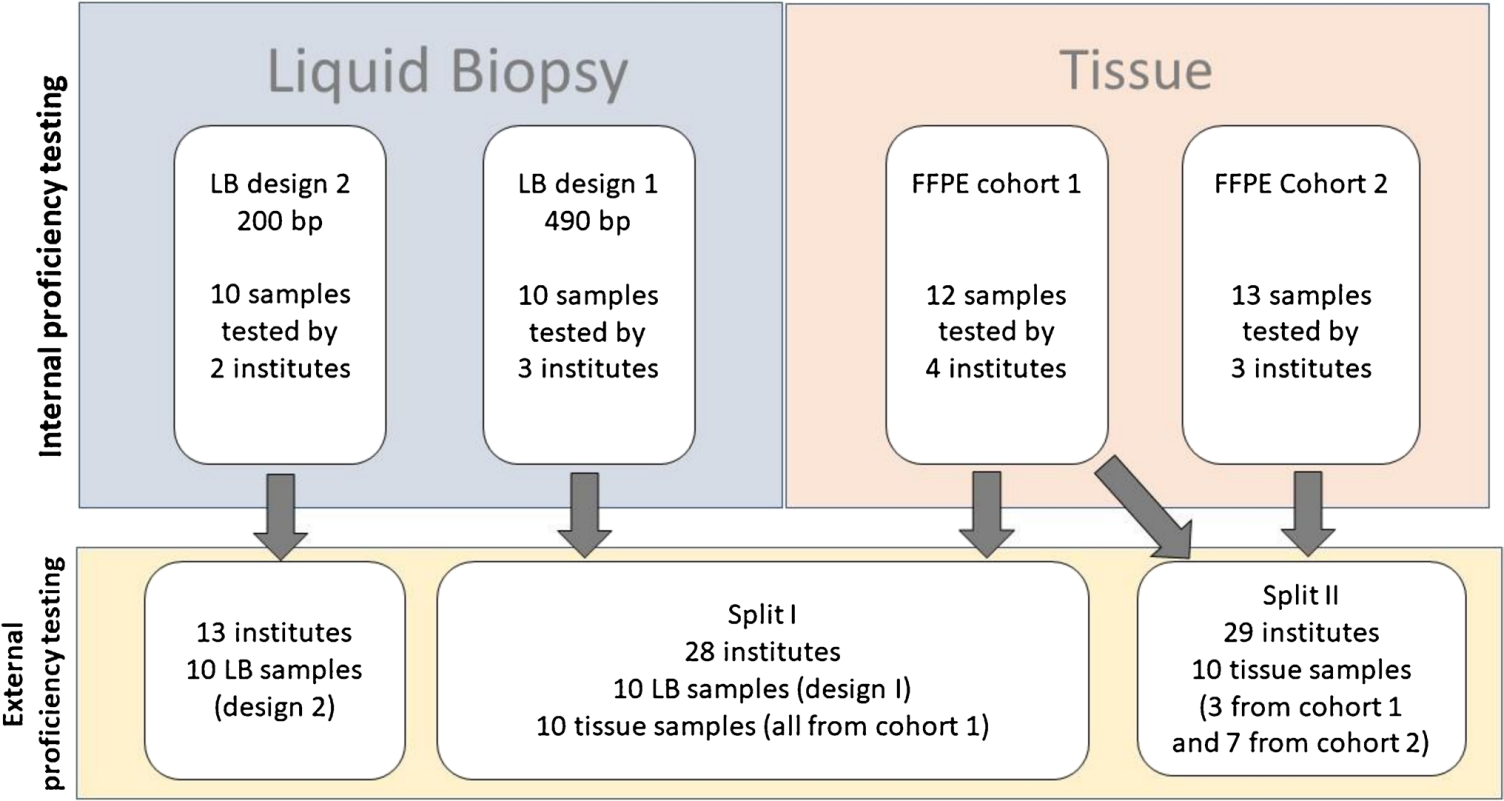
Summary of the set-up of the two phases described in detail in the material and methods section. First internal proficiency test was carried out to identify suitable samples and to define the consensus mutation status. The results of this internal proficiency test were used to assemble the sample cohorts for the ensuing external proficiency test with in total 62 institutions

A total of 25 HR+/HER2-*PIK3CA* mutation pretested breast cancer FFPE tissue samples with a minimal tumor cell content of 40%, were provided by five Institutes of Pathology (Charité-Universitätsmedizin Berlin, University Hospital Cologne, Technical University of Munich, Ludwig-Maximilians-Universität München, University of Regensburg) were used for the internal proficiency testing (see below) of which 20 were found to be suitable for external testing (Fig. 1).

#### Liquid biopsy

Samples composed of 2 ml DNA/RNA-free human plasma spiked with 60 ng fragmented DNA with defined allelic fractions of known mutations were sourced commercially.

The definition of reference values (ground truth) was based on the results of counter-testing by different panel institutes (internal proficiency testing). Cases for which > 25% of the participants evaluated a case incorrectly due to tumor heterogeneity or other reasons, were removed from the evaluation.

The external proficiency test was offered to German-speaking Institutes of Pathology. The participants had to submit their results within ten working days.

The preparation of all test sets and the conduct of the proficiency test was performed according to the specifications of DIN ISO EN 17043. The required quality management was executed by the QuIP office.

### Selection and internal proficiency testing of tissue samples

To ensure that the mutation status of the selected samples was representative and that the results were in line with the previous findings, 25 FFPE samples were split into two separated cohorts namely, split 1 (*n* = 12) and split 2 (*n* = 13) and re-evaluated by the Institute of Pathology, Charité-Universitätsmedizin Berlin and by three and two further institutes for split 1 and 2, respectively as part of an internal proficiency test using next generation sequencing (NGS), Sanger sequencing and quantitative real-time PCR (Suppl. Table 1). Therefore, three 4-μm thick unstained slides per case were provided to each participant, blinded to pretesting results.

Twenty samples with concordant results of at least three methods or three institutes were considered suitable for external proficiency testing. Following the re-evaluation, the sections for the external proficiency test were prepared. The last three sections of the test sets were again tested centrally at the Institute of Pathology at the Charité-Universitätsmedizin Berlin to ensure consistency throughout the FFPE block.

### Design and internal proficiency testing of liquid biopsy reference material

The LB reference samples were produced specifically by SensID (Rostock, Germany) and included *PIK3CA* hotspot mutations (NM_006218: Exon 9 c.1633G > A, p.E545K and Exon 20: c.3140A > G, p.H1047R) as single or double mutation with allelic fractions of 3% or 6% in a wild-type genomic DNA background. Additionally, all LB samples contained *KRAS* (NM_033360) Exon 2: c.35G > A, p.G12D mutated DNA fragments with an allelic fraction of 20% as an internal quality control. Two designs of LB test sets were constructed. Design I contained *PIK3CA* mutated DNA fragments of 490 bp length, in a background of significantly shorter *PIK3CA* wild-type fragments. The second design contained mutated fragments of around 167 bp length (± 10%) in a background of equally long wild-type fragments. The final test sets of design I and II contained five *PIK3CA* mutated and five *PIK3CA* wild-type samples.

Liquid biopsy reference samples (design I and II) were both tested centrally (Charité) with two different multiplex PCR-based NGS assays—LB-specific Oncomine Breast cfDNA assay (OBcfDNA, Thermo Fisher Scientific) and tissue-specific colon and lung version 2 panel (CLv2, Thermo Fisher Scientific) (Suppl. Table 2). The samples were counter-tested by two further institutes using either LB-specific clinical trial assay (therascreen) or hybrid capture-based assays.

### External proficiency testing

The external proficiency test was offered in two splits. Split 1 included the testing of 10 tissue and 10 LB samples of design I (long fragments). The second split included testing of 10 tissue samples. Liquid biopsy samples of design II (shorter fragments) could be tested additionally by the participants.

As defined in the internal proficiency test, participants received three 4-μm thick unstained sections per case for the tissue samples.

Liquid biopsy test sets were prepared as described above. For split 1 each part (tissue and LB) was scored separately with a maximum of 40 points each. Two points were awarded for each correctly diagnosed case namely the detection of the presence or absence of a treatable *PIK3CA* mutation. In addition, two points were given for the correct nomenclature of the mutations according to HGVS (human genome variation society). If a detection was not possible for technical reasons, one point was awarded (this option was available only once). Each part (tissue or LB) was considered to pass if they had ≥ 37 points, whereby ≥ 19 points had to be achieved for the first question (mutation: yes/no) and ≥ 18 points had to be achieved for the gene designation.

Participants received either a certificate confirming “successful participation” or a certificate of “participation” if the required passing score was not obtained.

## Results

This ring trial was conducted in two phases: firstly, an internal proficiency test with five participating laboratories was carried out to identify suitable samples and to define the consensus mutation status. The results of this internal proficiency test were used to assemble the sample cohort for the ensuing external proficiency test with in total 62 institutions (Fig. 1).

### Internal proficiency test—tissue

For internal testing of split 1 tissue, eight samples with concordant results of three institutes were used (Suppl. Table 3). Due to limited material, two *PIK3CA* mutated samples were used in duplicates. Tissue samples of split 1 contained six *PIK3CA* mutated (p.H1047R, p.E542K) and four *PIK3CA* wild-type samples. The results of the clinical trial qPCR assay (therascreen) showed strong divergence to expected values. The *PIK3CA* p.Q546R hotspot mutation was detected in 11 of 12 cases (split 1), which led to a false-positive rate of 92%. Therefore, results of this assay were excluded from further evaluation.

Seven samples tested for split 2 showed concordant results for at least three methods including *PIK3CA* mutations p.Q546K, p.E542K and p.H1047R (Suppl. Table 4). These samples and three wild-type samples tested in split 1 were considered suitable for external proficiency testing (10 samples split 1, 10 samples split 2). Allelic fractions of *PIK3CA* mutations ranged from 16 to 43%.

### Internal proficiency test—liquid biopsy

Testing of LB reference design I (long fragments) with NGS assays showed concordant results for *PIK3CA* and *KRAS* with unexpected low allelic fractions for the hybrid capture-based Avenio assay (min 0.1% to max 5.1% allelic fractions) (Suppl. Fig. 1).

One out of three institutes used the clinical trial assay (therascreen) showing false-positive *PIK3CA* p.Q546R mutations in three samples leading to a false-positive rate of 30%.

Liquid biopsy reference design II (short fragments) showed concordant results for LB-specific assays (OBcfDNA, therascreen, and AVENIO) with allelic fractions in the expected range (Suppl. Fig. 1). Regardless of the expected allelic fraction, *PIK3CA* p.H1047R and *KRAS* p.G12D mutations were not detectable with the tissue-specific CLv2 assay in design II.

### External proficiency testing

In total, 62 institutions from four countries registered for the external proficiency test, including 51 participants from Germany, eight from Austria, two from Switzerland, and one from Italy. Twenty-eight were applied for split 1 (ten tissue and ten LB samples) and 29 for split 2 (10 tissue samples). Seven institutes, which participated in split 1, and six additional institutes also opted in for LB testing of design II.

### External proficiency testing—tissue

Forty-three of fifty-four participants (80%, split 1 and split 2) submitted results and 43 participants (80%) successfully passed the trial (Table 1).

**Table 1:** Methods and results of external proficiency tissue test split 1 and 2; NGS, next generation sequencing; qPCR, quantitative polymerase chain reaction; ddPCR, digital droplet PCR

Methods	Number of participants [*n* = 54]	Successful [*n* = 43]
NGS	34 (63%)	28 (82.4%)
Sanger sequencing	7 (13%)	4 (57.1%)
Mutation/allele-specific qPCR	4 (8%)	3 (75%)
Pyrosequencing	3 (6%)	3 (100%)
NGS and mutation/allele-specific qPCR	2 (4%)	2 (100%)
PCR and mass spectrometry	1 (2%)	1 (100%)
Sanger sequencing and mutation/allele-specific qPCR	1 (2%)	1 (100%)
Sanger sequencing and NGS	1 (2%)	1 (100%)
ddPCR	1 (2%)	0 (0%)

For split 1, one wild-type sample was not evaluable in 5 cases and false-positive in two cases, which constituted > 25% (7/27) of the participants. Therefore, participants were given full scores for this case, and it was excluded from further evaluation.

For split 2, one *PIK3CA* p.Q546K mutated case was reported as false-negative by > 25% of the participants (7/27). Therefore, according to predefined definitions for the evaluation phase, the case was also excluded from further consideration. All attendees were given full scores for this case, and it was excluded from further analysis. Altogether, *PIK3CA* mutation detection for tissue reached a sensitivity > 98% and specificity of 97%, kappa 0.92 (Table 2).

**Table 2:** Results from 54 participants (27 split 1, 27 split 2) of external proficiency testing. Data include results of 18 tissue samples (split 1: 6× *PIK3CA* mutated (mut), 3× wild-type (WT), split 2: 6× *PIK3CA* mut, 3× WT). Two samples (1× WT split 1, 1× *PIK3CA* mut split 2) were excluded due > 25% deviation from external vs. internal results

		*PIK3CA* mut [*n* = 351]	*PIK3CA* WT [*n* = 135]
Results external testing (tissue)	*PIK3CA* mut	338/351 (96.3%)	4/135 (3.0%)
	*PIK3CA* WT	5/351 (1.4%)	129/135 (95.6%)
	Not analyzable	5/351 (1.4%)	5/135 (3.7%)
	Cohen`s Kappa	0.92	
	Sensitivity	98.5%	
	Specificity	97.0%	

### External proficiency testing—liquid biopsy

Eighty-three percent of the participants (20/24) that analyzed LB design I and 31% (4/13) for design II passed the proficiency testing successfully. The sensitivity of *PIK3CA* mutation detection was higher for participants using design I (95.8% vs. 73.0%). The same was observed for the specificity (98.3% vs. 82.1%) and kappa value (0.94 vs 0.55) (Table 3).

**Table 3:** Results of liquid biopsy external proficiency testing from 24 (design I, mutated fragments around 490 bp length) and 13 participants (design II, mutated fragments around 167 bp length). Data include results of 10 liquid biopsy samples (5× *PIK3CA* mutated (mut), 5× wild-type (WT))

			*PIK3CA* mut		*PIK3CA* WT	
			Design I [*n* = 120]	Design II [*n* = 65]	Design I [*n* = 120]	Design II [*n* = 65]
Results external testing (liquid biopsie)	*PIK3CA* mut		114/120 (95.0%)	46/65 (70.7%)	2/120 (1.7%)	10/65 (15.4%)
	*PIK3CA* WT		5/120 (4.2%)	17/65 (26.1%)	117/120 (97.5%)	46/65 (69.2%)
	Not analyzed		1/120 (0.8%)	2/65 (3.1%)	1/120 (0.8%)	8/65 (1.5%)
	Sensitivity	Design I (490bp)	95.8%			
		Design II (167bp)	73.0%			
	Specificity	Design I (490bp)	98.3%			
		Design II (167bp)	82.1%			
	Cohen`s Kappa	Design I (490bp)	0.94			
		Design II (167bp)	0.55			

Four participants did not pass the LB testing part with reference design I. One used a tissue-specific NGS assay (CLv2, Thermo Fisher Scientific) and had a false-negative result regarding a *PIK3CA* p.E545K mutation with an allelic fraction of 3%. Additionally, one participant evaluated a p.H1047R *PIK3CA* mutated sample with an allelic fraction of 3% as false-negative and two wild-type samples as false-positive, reporting *PIK3CA* p.C420R (1.9% allelic fraction) and a hotspot mutation p.E542K (0.9% allelic fraction).

Nine participants did not pass the testing of reference design II, four of them used a LB-specific assay. Results of mutation detection with design II showed a higher false-positive rate (15.4% vs. 1.7%) and a higher false-negative rate (26.1% vs. 4.2%). Participants who passed the trial used either digital droplet PCR (ddPCR), PCR/mass spectrometry, or LB-specific NGS assays.

**Fig 2:**
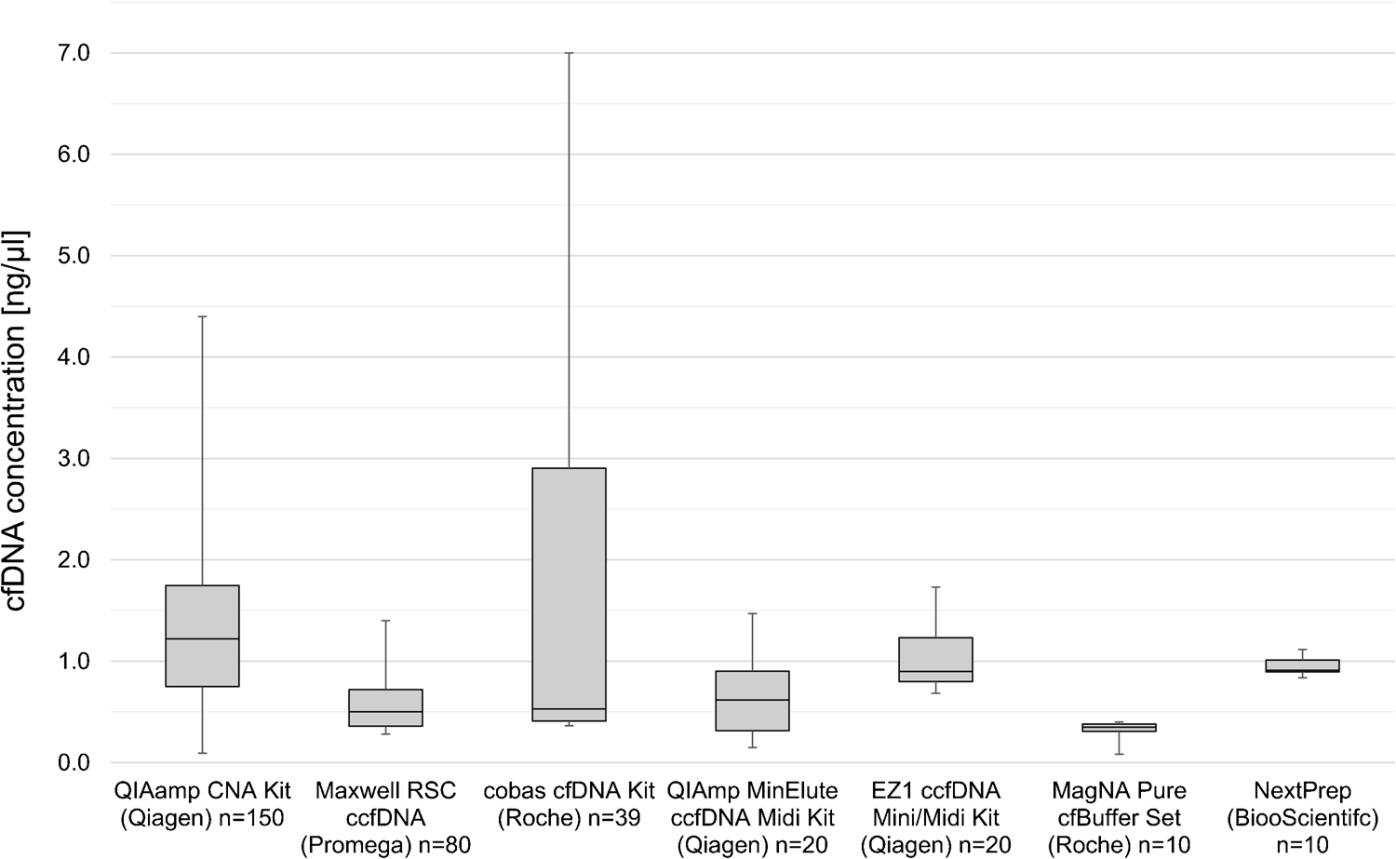
Range of cfDNA concentrations related to extraction methods used for design I and II. Results include data from 33 participants. Two participants did not report concentrations (design I), one was incorrect and data of spectroscopic measurement was excluded because of high divergence. CNA, circulating nucleic acid

cfDNA concentration was available from 35 participants and for all ten LB test sets of design I and II (Fig. 2). Two participants did not report the concentration, one reported constantly 10 ng/μl for all cases, and one participant used spectroscopic measurement leading to significant higher concentrations of more than 6 ng/μl. These data were excluded from further analysis. The highest concentrations were achieved when using a column-based silica membrane kit (QIAamp circulating nucleic acid kit) for cfDNA extraction with median of 1.22 ng/μl (range 0.25–4.40).

**Fig 3:**
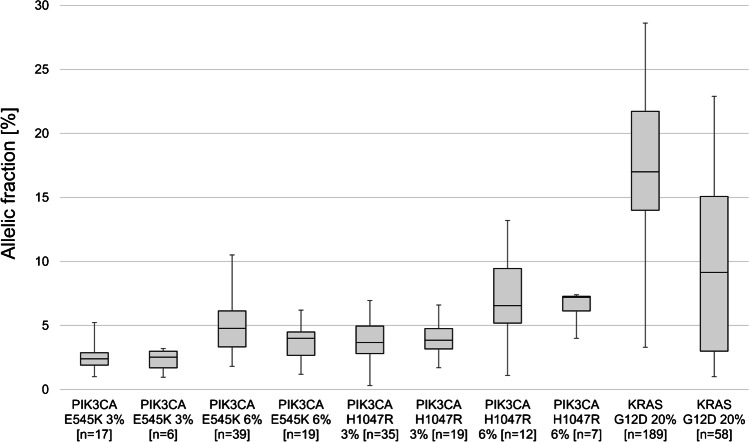
Reported allelic fraction for PIK3CA and KRAS mutations in liquid biopsy reference samples design I (white) and design II (grey)

Depending on the method, allelic fractions were reported by 21/24 participants for design I and 11/13 for design II. *PIK3CA* p.E545K mutation reference with 3% allelic fraction was detected in a range of 1 to 5% (median 2.4%) for design I vs. 1 to 3% (median 2.5%) for design II (Fig. 3). The p.E545K 6% reference was detected in a range from 2 to 11% allelic fraction (median 4.8%) vs. 1 to 6% (median 4.0%) for design I and II, respectively. *PIK3CA* p.H1047R mutation reference with 3% allelic fraction was detected in a range of 0.3 to 7% (median 3.6%) with an outlier at 32% allele frequency, which was excluded from the analysis. Design II showed a smaller range of 2 to 7% (median 3.9%). The *PIK3CA* p.H1047R 6% reference was detected in a wide range from 1 to 13% allelic fraction (median 7.0%) for design I vs. 4 to 7% (median 7%) for design II. *KRAS* p.G12D 20% allelic fraction reference was reported in a much higher range of 3 to 29% (median 17.0%) for design I vs. 1 to 23% (median 9.2%) for design II. A constantly low allelic fraction of 1 to 2% for all ten LB design II samples was reported with tissue-specific OFA (Thermo Fisher Scientific). The highest values around 15% were achieved with LB-specific Oncomine Breast cfDNA assay (Thermo Fisher Scientific).

**Fig 4:**
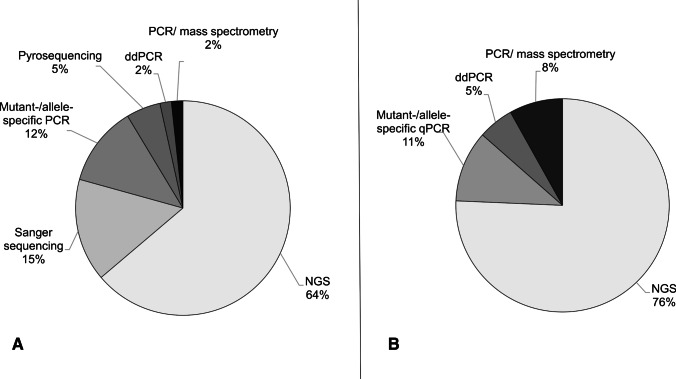
Methods used for mutation detection in external proficiency testing. **A** Methods used for tissue testing (split 1 and 2) [*n* = 58]. **B** Methods used for liquid biopsy analysis (design I and II) [*n* = 38]; NGS, next generation sequencing; ddPCR, digital droplet PCR

The most commonly used method for determination of *PIK3CA* mutation status was NGS for both, tissue (34/54 participants) and LB (18/24 participants) analyses (Fig. 4).

**Fig 5:**
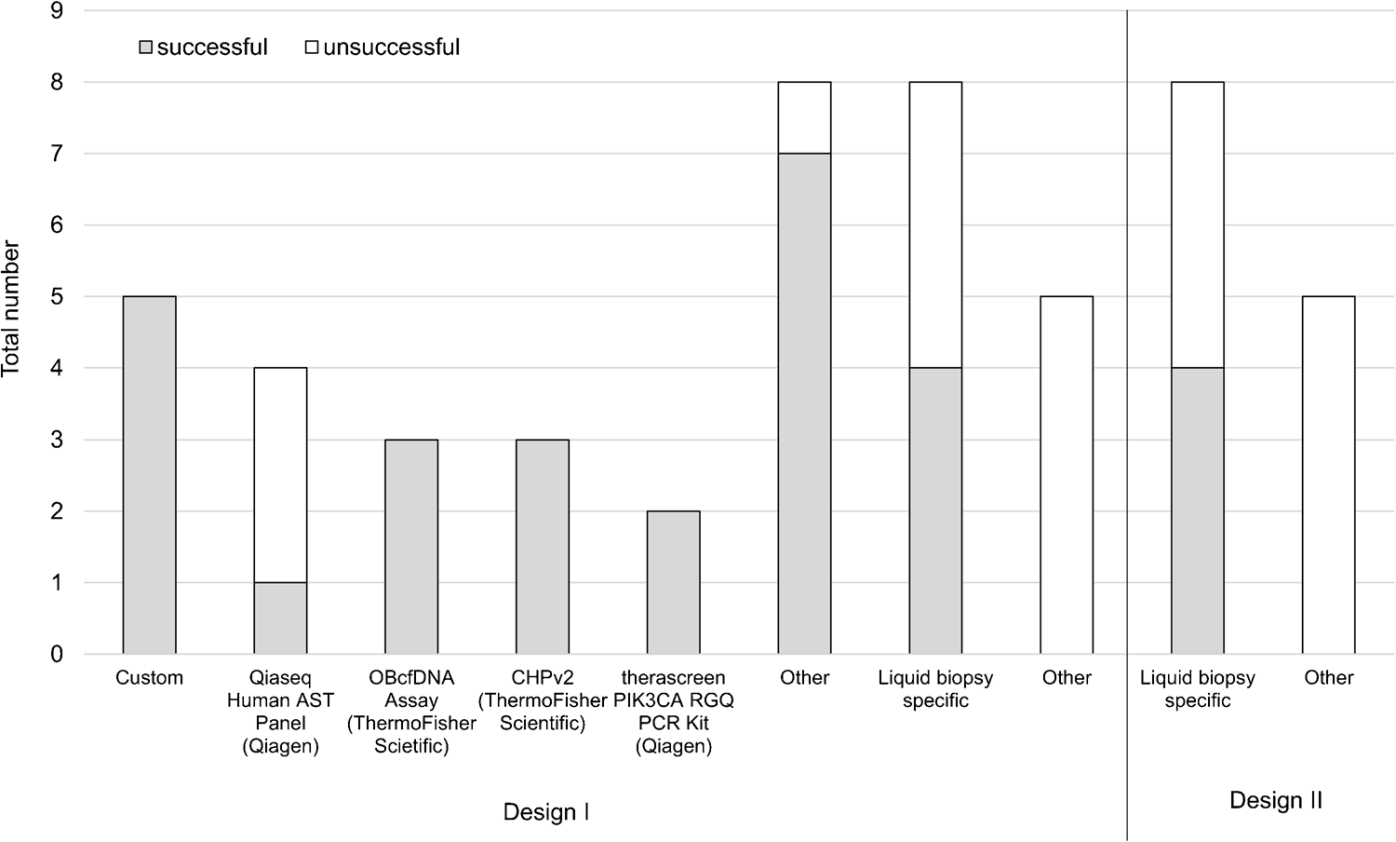
Most commonly used assays for liquid biopsy mutation testing (design I and II). AST, actionable solid tumor; OBcfDNA, Oncomine breast cfDNA; CHPv2, cancer hotspot panel version 2

The participants used a broad range of assays for *PIK3CA* mutation analyses (Fig. 5). Five participants evaluating LB design I who used customized assays, including NGS amplicon-based panel and primer for ddPCR, passed the trial. One participant who did not pass the proficiency test used amplicon-based NGS assays specified for tissue specimens. Two participants successfully applied the clinical trial assay (therascreen) used in the SOLAR-1 trial for molecular stratification of activating *PIK3CA* mutations. All participants who tested LB design II and used a non-specific LB assay did not pass the trial.

## Conclusion

For the identification of breast cancer patients who might benefit from targeted therapy with specific PIK3CA inhibitors, locally deployable highly sensitive, and specific *PIK3CA* mutation testing that covers all actionable mutations is required. Proficiency testing is an appropriate approach to assess the reliability of diagnostic centers for molecular detection of *PIK3CA* mutations. In Germany, the Quality in Pathology-QuIP GmbH offers proficiency testing in pathology. In cooperation with selected (lead) panel institutes, proficiency testing for new molecular targets is established or repeatedly performed for already established targets.

With the recent approval of a specific PIK3CA inhibitor for breast cancer patients whose tumors harbor *PIK3CA* mutations or have *PIK3CA* mutations in their ctDNA, the proficiency testing for *PIK3CA* mutation analysis was initiated both for tissue and LB samples. The spectrum of *PIK3CA* mutations which was included in the proficiency testing was in line with the approved study [13]. Whereas the mutation analysis in tissue samples is well-established for many different alterations, proficiency testing for LB is a relatively new area within the QuIP portfolio [14, 15].

Thus, the results of the ring trial for the tissue samples bear few surprises. Sourcing adequate samples were somewhat of a challenge. Firstly, large tissue samples are frequently derived from resections that may have relatively long formalin fixation times leading to an accumulation of DNA artifacts. Secondly, the vast majority of cases carried a *PIK3CA* mutation at position p.H1047R. Here, care was taken to cover a range of actionable *PIK3CA* variants to ensure optimal stratification of patients.

With an allelic fraction of *PIK3CA* mutations ranging from 16 to 43%, Sanger sequencing was expected to be at the limit of detection. This is consistent with only four of seven (57.1%) centers using Sanger sequencing to pass the external ring trial compared to centers using NGS (82.2%). That Sanger sequencing is of limited sensitivity in samples with relatively low tumor content is well established and reflects why it was only used by 13% of the participating centers. Sequencing-based methods including Sanger sequencing or NGS have the advantage over RT-PCR-based methods that the variants to be detected do not need to be predefined at the time of the assay design. Martínez-Sáez et al*.*, for example, showed that nearly one-third of *PIK3CA* mutations identified in a cohort of HR+/HER2-breast cancer patients were not covered by the clinical trial assay [16].

Setting up a ring trial for LB assays proved to be more challenging; however, in order to produce well defined and reproducible testing conditions, human plasma devoid of nucleic acids was supplemented by fragmented wild-type genomic DNA and additionally spiked with synthetic DNA fragments harboring *PIK3CA* mutations of defined allelic fractions.

These LB reference samples were designed and manufactured by a commercial vendor. However, the results of internal proficiency test of the initial design (I) showed unexpected low allelic fractions when analyzed by a hybrid capture assay. This could be explained by the fact that the mutated DNA fragments were approximately 490 bp long and considerably longer than the wild-type DNA counterparts. This led to a preferential enrichment of the shorter wild-type fragments. Thus, a second design with mutated *PIK3CA* fragments of comparable length to the wild-type fragments of (approx. 167 bp) was provided (design II).

The internal proficiency testing based on design II provided allelic fractions in the expected range with the hybrid capture enrichment approach. Although the longer *PIK3CA* fragments (design I) technically provide a better template for amplicon-based NGS assays, this did not reflect cfDNA fragments in clinical LB very well. Consequently, amplicon-based NGS assays not specifically designed for the detection of short cfDNA fragments in LB underperformed in this proficiency test using design II.

Reference samples with long DNA fragments (design I) were provided to all participants not using hybrid capture-based methods—well aware of the fact that this was not equivalent to clinical samples. For transparency, this fact was pointed out in the certificates with the statement “Liquid Biopsy—with mutated fragments of 490 bp.”

After external proficiency testing, the methods used by the participants were reviewed for general eligibility for LB testing by the lead panel institute. Seven of the participants already applied an assay specifically designed for the use with cfDNA from plasma samples. These institutes were also offered participation in the proficiency testing scheme employing design II. All participants that did not use LB-specific assays for testing of reference samples with design II failed, which might be one explanation for the high false-negative rate (26%). In addition, false-positive results occurred using the original clinical trial assay (therascreen) during the internal proficiency testing. This was addressed by a press release of the assay provider [17].

Since a broad range of assays was used in the present ring trial, including RUO assays often with protocols adjusted to local requirements, the results here cannot provide general performance matrices on the different assays. To compare assays regarding their sensitivity, specificity, and limit of detection, the performance characteristics of a given assay might be inquired for further ring trials upon registration. From the data presented here we cannot draw any conclusions on the level of certain assays but once more, our data clearly show that Sanger sequencing is not suitable for liquid biopsy testing.

In conclusion, the use of well-designed standardized reference material becomes more important and even necessary considering the growing spectrum of biomarkers and materials that needs to be evaluated. Besides the striking advantages such as quality conformance, standardized conditions, flexibility in design, reproducibility, and preservation of sometimes rare patient material the presented data also underline the complexity of the implementation. Very careful consideration of the design—not only of the LB reference samples—is essential in order to cover the full range of assays applicable to *PIK3CA* mutation detection.

## Supplementary Information

Suppl. Table 1 (DOCX 13 kb)

Suppl. Table 2 (DOCX 12 kb)

Suppl. Table 3 (DOCX 14 kb)

Suppl. Table 4 (DOCX 14 kb)

Suppl. Fig 1 Allelic fractions of PIK3CA and KRAS mutations of internal proficiency testing for liquid biopsy design I (left bar) and design II (right bar); OBcfDNA: Oncomine Breast cfDNA assay – liquid biopsy specific (Thermo Fisher Scientific), CLv2: Colon and Lung version 2 panel - FFPE specific (Thermo Fisher Scientific), AVENIO ctDNA assay – liquid biopsy specific (Roche), n/d: not detected. (PNG 145 kb)

High Resolution Image (EPS 14637 kb)

## References

[1] Smith RA (2018). Cancer screening in the United States, 2018: a review of current American Cancer Society guidelines and current issues in cancer screening. CA Cancer J Clin.

[2] Rosenbaum JN, Weisman P (2017). The evolving role of companion diagnostics for breast cancer in an era of next-generation omics. Am J Pathol.

[3] Howlader N et al (2014) US incidence of breast cancer subtypes defined by joint hormone receptor and HER2 status. J Natl Cancer Inst 106(5):dju055 10.1093/jnci/dju055 PMC4580552 24777111

[4] Setiawan VW (2009). Breast cancer risk factors defined by estrogen and progesterone receptor status: the multiethnic cohort study. Am J Epidemiol.

[5] Jhaveri K et al (2021) The evolution of cyclin dependent kinase inhibitors in the treatment of cancer. Expert Rev Anticancer Ther 10.1080/14737140.2021.1944109 PMC12592983 34176404

[6] Goncalves MD, Hopkins BD, Cantley LC (2018). Phosphatidylinositol 3-kinase, growth disorders, and cancer. N Engl J Med.

[7] Cancer Genome Atlas N (2012). Comprehensive molecular portraits of human breast tumours. Nature.

[8] Janku F (2017). Phosphoinositide 3-kinase (PI3K) pathway inhibitors in solid tumors: from laboratory to patients. Cancer Treat Rev.

[9] Gymnopoulos M, Elsliger MA, Vogt PK (2007). Rare cancer-specific mutations in PIK3CA show gain of function. Proc Natl Acad Sci U S A.

[10] Bader AG (2005). Oncogenic PI3K deregulates transcription and translation. Nat Rev Cancer.

[11] Gao J (2013). Integrative analysis of complex cancer genomics and clinical profiles using the cBioPortal. Sci Signal.

[12] Cerami E (2012). The cBio cancer genomics portal: an open platform for exploring multidimensional cancer genomics data. Cancer Discov.

[13] Andre F (2019). Alpelisib for PIK3CA-mutated, hormone receptor-positive advanced breast cancer. N Engl J Med.

[14] Fassunke J (2017). EGFR T790M mutation testing of non-small cell lung cancer tissue and blood samples artificially spiked with circulating cell-free tumor DNA: results of a round robin trial. Virchows Arch.

[15] Jurmeister P (2021). Status quo of ALK testing in lung cancer: results of an EQA scheme based on in-situ hybridization, immunohistochemistry, and RNA/DNA sequencing. Virchows Arch.

[16] Martinez-Saez O (2020). Frequency and spectrum of PIK3CA somatic mutations in breast cancer. Breast Cancer Res.

[17] QIAGEN, Dringende sicherheitsinformation, in therascreen® PIK3CA RGQ PCR Kit, REF 873111. 2020, QIAGEN. p. 4.

