# Supplementary material for: Proficiency testing of PIK3CA mutations in HR+/HER2-breast cancer on liquid biopsy and tissue
Source: Virchows Arch. 2022 Nov 11;482(4):697–706. doi: 10.1007/s00428-022-03445-x (PMC10067656; doi:10.1007/s00428-022-03445-x)
Supplement: Supplementary file 1 — (DOCX 13 kb) [file 428_2022_3445_MOESM1_ESM.docx]

Suppl. table 1: Methods used for tissue internal proficiency testing; OFA: Oncomine Focus Assay, FFPE: formalin-fixed paraffin-embedded tissue

|  | **Participant** | | | | |
| --- | --- | --- | --- | --- | --- |
|  | **Lead** | **Panel 1** | **Panel 2** | **Panel 3** | **Panel 4** |
| DNA  extraction | Maxwell RSC DNA FFPE Kit (Promega) | Maxwell RSC DNA FFPE Kit (Promega) | QIAamp DSP DNA FFPE Tissue Kit (Qiagen) | Maxwell RSC DNA FFPE Kit  (Promega) | GeneRead DNA FFPE Kit  (Qiagen) |
| DNA  quantification | Fluorometric | qPCR | Fluorometric | Spectro-photometric | Fluorometric |
| Method | Ion S5  (Themo Fisher Scientific) | MiSeq/ NextSeq (Illumina) | Allele specific PCR  Rotor-Gene Q (Qiagen)  &  Illumina | PCR & Sanger-Sequencing  (Genetic Analyzer, Thermo Fisher Scientific) | Ion S5/PGM (Thermo FisherScientific) &  PyroMark (Qiagen) |
| Assay | OFA (ThermoFisher Scientific) | GeneRead Custom Panel (Qiagen) | therascreen PIK3CA RGQ PCR Kit (Qiagen)  &  TruSight Tumor15 Panel (Illumina) | Customized | OFA  (Thermo Fisher Scientific) |
| Split | 1, 2 | 1 | 1, 2 | 1 | 2 |
