# Supplementary material for: Proficiency testing of PIK3CA mutations in HR+/HER2-breast cancer on liquid biopsy and tissue
Source: Virchows Arch. 2022 Nov 11;482(4):697–706. doi: 10.1007/s00428-022-03445-x (PMC10067656; doi:10.1007/s00428-022-03445-x)
Supplement: Supplementary file 2 — (DOCX 12 kb) [file 428_2022_3445_MOESM2_ESM.docx]

Suppl. table 2: Methods used for liquid biopsy internal proficiency testing; OBcfDNA: Oncomine Breast cfDNA Assay, CLv2: Colon and Lung Version 2 Panel

|  | **Participants** | | |
| --- | --- | --- | --- |
|  | **Lead** | **Panel 1** | **Panel 2** |
| DNA  extraction | Maxwell ccfDNA Kit (Promega) | QIAamp DSP Circulating NA Kit (Qiagen) | Maxwell ccfDNA Kit (Promega) |
| DNA  quantification | Fluorometric  (Quantus, Promega) | Fluorometric | Not performed |
| Method | Ion S5  (Thermo Fisher Scientific) | Rotor-Gene Q (Qiagen) | MiSeq/NextSeq (Illumina) |
| Assay | OBcfDNA & CLv2  (Thermo Fisher Scientific) | therascreen PIK3CA RGQ PCR Kit (Qiagen) | AVENIO ctDNA targeted Kit (Roche) in combination with Illumina baits |
