# Supplementary material for: Proficiency testing of PIK3CA mutations in HR+/HER2-breast cancer on liquid biopsy and tissue
Source: Virchows Arch. 2022 Nov 11;482(4):697–706. doi: 10.1007/s00428-022-03445-x (PMC10067656; doi:10.1007/s00428-022-03445-x)
Supplement: Supplementary file 3 — (DOCX 14 kb) [file 428_2022_3445_MOESM3_ESM.docx]

Suppl. table 3: Results of internal proficiency testing – tissue, split 1; Mut: *PIK3CA* mutation; AF: allelic fraction; WT: wild-type; NA: not applicable. *therascreen PIK3CA PCR Assay (Qiagen); **TruSight Tumor15 Panel (Illumina); *** endpoint testing lead institute. Deviations are written italic.

|  | | **Lead** | | **Panel 1** | | **Panel 2** | | | | **Panel 3** | |
| --- | --- | --- | --- | --- | --- | --- | --- | --- | --- | --- | --- |
| **Internal testing** | **Selected**  **for external testing** | **Mut** | **AF**  **[%]** | **Mut** | **AF**  **[%]** | **Mut*** | **AF**  **[%]*** | **Mut**** | **AF**  **[%]**** | **Mut** | **AF**  **[%]** |
| 1 | 5/8 | H1047R | 22 | H1047R | NA | *Q546R*  H1047R | NA | H1047R | 19 | H1047R | NA |
| 2 | - | WT | - | WT | - | *Q546R* | NA | WT | - | WT |  |
| 3 | 9 | H1047R | 28 | H1047R | NA | *Q546R*  H1047R | NA | H1047R | 29 | H1047R | NA |
| 4 | 3 | WT | - | WT | - | *Q546R* | NA | WT | - | WT | - |
| 5 | 10 | WT | - | WT | - | *Q546R* | NA | WT | - | WT | - |
| 6 | - | WT | - | WT | - | *Q546R* | NA | WT | - | WT | - |
| 7 | 6 | WT | - | WT | - | WT | - | WT | - | WT | - |
| 8 | 4/7 | E542K | 28 | E542K | NA | E542K  *Q546R* | NA | E542K | 20 | E542K | NA |
| 9 | 1 | E542K | 43 | E542K | NA | E542K  *E545K* | NA | E542K  *E545K* | 5;  23 | E542K | NA |
| 10 | - | E542K  E545K*** | 23 | E542K | NA | E542K  *Q546R* | NA | E542K | 15 | E542K | NA |
| 11 | - | E542K | 19 | E542K | NA | E542K  *Q546R* | NA | E542K | 19 | E542K | NA |
| 12 | 2 | E542K | 22 | E542K | NA | E542K  *Q546R* | NA | E542K | 20 | E542K | NA |
