# Supplementary material for: Proficiency testing of PIK3CA mutations in HR+/HER2-breast cancer on liquid biopsy and tissue
Source: Virchows Arch. 2022 Nov 11;482(4):697–706. doi: 10.1007/s00428-022-03445-x (PMC10067656; doi:10.1007/s00428-022-03445-x)
Supplement: Supplementary file 4 — (DOCX 14 kb) [file 428_2022_3445_MOESM4_ESM.docx]

Suppl. table 4: Results of internal proficiency testing – tissue, split 2; Mut: *PIK3CA* mutation; AF: allelic fraction; WT: wild-type; NA: not applicable; deviations are written italic.

|  | | **Lead** | | **Panel 2** | | | | **Panel 4** | | | |
| --- | --- | --- | --- | --- | --- | --- | --- | --- | --- | --- | --- |
| **Internal**  **testing** | **Selected**  **for external testing** | **Mut** | **AF**  **[%]** | **Mut** | **AF**  **[%]** | **Mut** | **AF**  **[%]** | **Mut** | **AF**  **[%]** | **Mut** | **AF**  **[%]** |
| 1 | 1 | Q546K | 16 | *E545D* | NA | *WT* | - | Q546K | NA | Q546K | NA |
| 2 | 2 | H1047K | 43 | H1047R | NA | - | - | H1047R | NA | - | - |
| 3 | 5 | E542K | 19 | E542K | NA | - | - | E542K | NA | - | - |
| 4 | 8 | E542K | 26 | E542K | NA | - | - | E542K | NA | - | - |
| 5 | - | E545K | NA | E545K | NA | E545K | NA | --- | - | H1047R (weak); E545K (very weak) | NA |
| 6 | 4 | E542K | 29 | E542K | NA | - | - | E542K | NA | - | - |
| 7 | - | E542K | 14 | E542K | NA | - | - | E542K | NA | - | - |
| 8 | - | E542K | NA | E545K | NA | E545K | NA | --- | - | WT | NA |
| 9 | 9 | E542K | 26 | E542K | NA | - | - | E542K | NA | - | - |
| 10 | - | WT | - | *H1047R* | NA | WT | - | *H1047R* | NA | WT | NA |
| 11 | - | E542K | 53 | E542K | NA | - | - | E542K | NA | - | - |
| 12 | 7 | E542K | 25 | E542K | NA | - | - | E542K | NA | - | - |
| 13 | - | H1047R | 43 | H1047R | NA | - | - | H1047R | NA | - | - |
