# Supplementary figures and images for: Proficiency testing of PIK3CA mutations in HR+/HER2-breast cancer on liquid biopsy and tissue
Source: Virchows Arch. 2022 Nov 11;482(4):697–706. doi: 10.1007/s00428-022-03445-x (PMC10067656; doi:10.1007/s00428-022-03445-x)

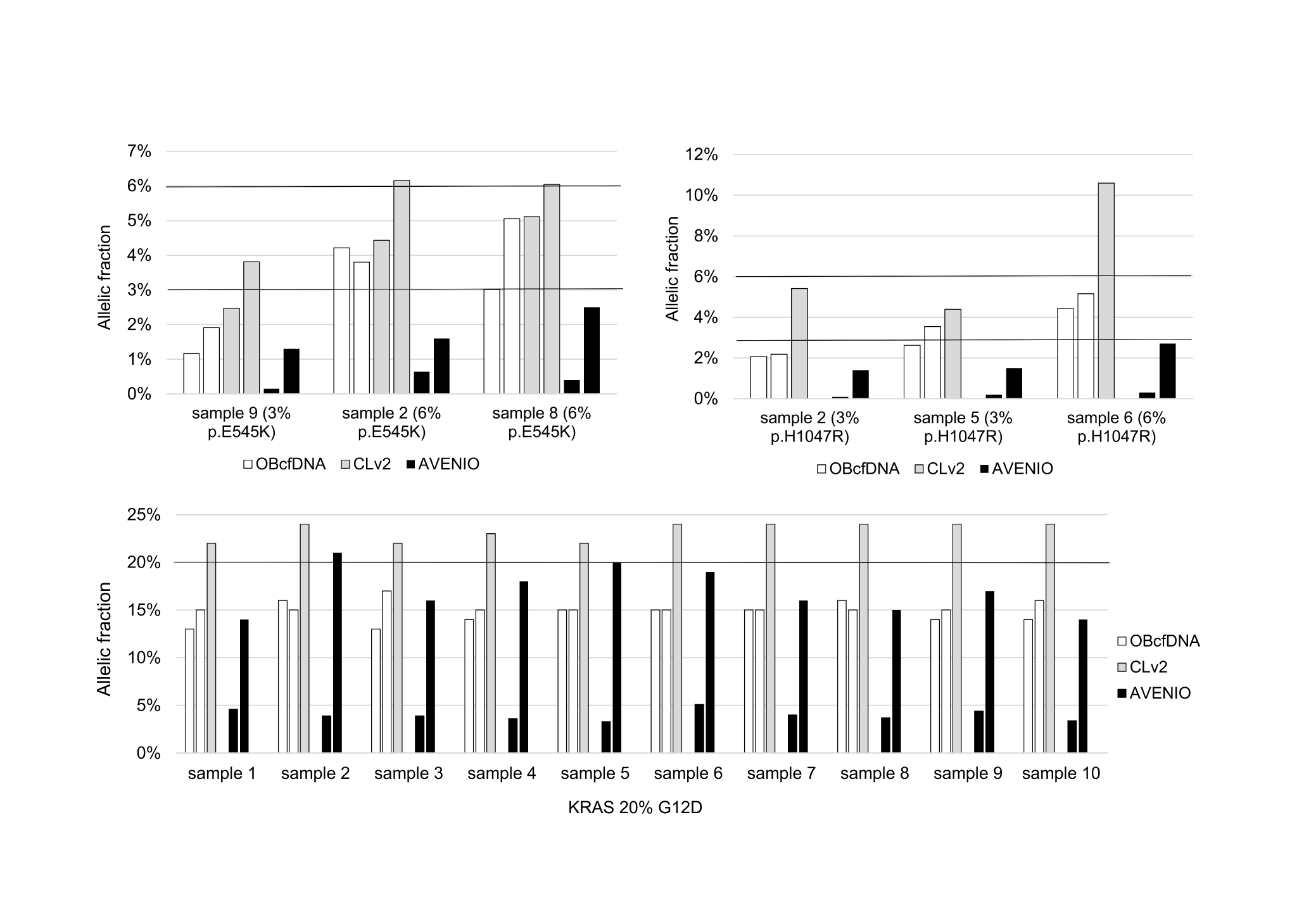

Supplement: Supplementary file 5 — Allelic fractions of PIK3CA and KRAS mutations of internal proficiency testing for liquid biopsy design I (left bar) and design II (right bar); OBcfDNA: Oncomine Breast cfDNA assay – liquid biopsy specific (Thermo Fisher Scientific), CLv2: Colon and Lung version 2 panel - FFPE specific (Thermo Fisher Scientific), AVENIO ctDNA assay – liquid biopsy specific (Roche), n/d: not detected. (PNG 145 kb) [file 428_2022_3445_Fig6_ESM.png]
